# IOLMaster versus Manual Keratometry after Photorefractive Keratectomy

**Published:** 2011-07

**Authors:** Hasan Razmju, Leila Rezaei, Kobra Nasrollahi, Hamid Fesharaki, Hossein Attarzadeh, Farhad Janbaz Footami

**Affiliations:** Isfahan Eye Research Center, Feiz Hospital, Isfahan University of Medical Sciences, Isfahan, Iran

**Keywords:** Keratometry, IOLMaster, Javal, Clinical History Method

## Abstract

**Purpose:**

To compare keratometric measurements using a Javal type manual keratometer with IOLMaster in eyes undergoing photorefractive keratectomy (PRK) for myopia.

**Methods:**

In this comparative case series, we studied patients aged 21 to 27 years scheduled for myopic PRK. Keratometry was performed preoperatively and three months after the procedure using a Javal type manual keratometer and the IOLMaster. We compared postoperative measurements obtained by both instruments with the clinical history method (CHM).

**Results:**

Seventy eyes of 35 patients with mean age of 23.45±1.55 years were studied. Mean preoperative spherical equivalent was −4.53±1.3 D. Average preoperative IOLMaster and manual keratometric readings were 45.95±1.23 D and 46.32±1.18 D, respectively. Postoperatively, mean IOLMaster measurements was 38.03±0.68 D and that of manual keratometry was 43.15±1.1 D. Compared to CHM measurements, the 95% limits of agreement were −5.95 to −0.85 for the IOLMaster and −1.44 to 4.04 for manual keratometry.

**Conclusion:**

Keratometric measurements with the IOLMaster and a Javal type manual device are comparable after PRK; both are largely deviant from the CHM and can yield misleading results.

## INTRODUCTION

Reduced accuracy of intraocular lens (IOL) calculations following keratorefractive surgery is a clinical problem of growing importance.^1^ Experience with eyes after myopic laser ablation indicates that incorporation of average keratometric readings into standard IOL power formulas frequently results in substantial under-correction and hyperopia/anisometropia following cataract surgery, depending on the amount of previously corrected myopia.

To avoid hyperopia after cataract surgery, eyes with previous keratorefractive surgery should be evaluated with a variety of measurement techniques to determine the keratometric value around which results tend to cluster, thus leading to less erroneous IOL calculations.^2^,^3^ In eyes which keratometry and refraction prior to keratorefractive surgery are available, the gold standard is to subtract the observed change in spherical equivalent (SE) at the corneal plane from preoperative central keratometric power. However, if this data is not available, standard keratometric readings could result in hyperopia.^4^ The major source of error in IOL power calculation after keratorefractive surgery is relying on inaccurate measurements obtained by corneal topographic systems and keratometers.

Currently, different keratometers are in use with varying levels of accuracy. Studies have reported conflicting results regarding the superiority of certain keratometers or that measurements performed with the IOLMaster^5^ or Pentacam^6^ can be used with sufficient accuracy.

In this study, we compare a manual Javal type keratometer with the IOLMaster for obtaining keratometric measurements in eyes undergoing photorefractive keratectomy (PRK) and compare their accuracy with the clinical history method (CHM).

## METHODS

This comparative case series included 70 eyes of 35 patients scheduled for myopic PRK. A skilled operator familiar with both instruments collaborated with the study and keratometry was performed with the IOLMaster (Carl Zeiss, Jena, Germany) and a manual Javal type keratometer (Gm 300; CSO, Milano, Italy), one day before surgery. Manual keratometry was performed first, followed by IOLMaster keratometry after 5 to 10 minutes. Patient data including age, sex, refractive error as determined by autorefraction (Topcon 750i; Topcon, Tokyo, Japan), and keratometric readings were recorded. Tissue-saving excimer laser PRK was performed the following day (Z100; Technolas, Munich, Germany).

Three months after the procedure, IOLMaster measurement, manual keratometry and autorefraction were repeated. According to the CHM, postoperative corneal power was calculated by subtracting the change in manifest refraction at the corneal plane induced by PRK from preoperative corneal keratometric values obtained by each keratometer prior to surgery.^7^ Postoperative measurements obtained by each device were compared to the CHM and results were compared.

### Statistical Analysis

Descriptive data are presented as mean ± SD with range. Paired differences between keratometric measurements and CHM-derived keratometry are presented as mean differences, 95% confidence intervals (CIs), and 95% limits of agreement (LoA). The 95% LoA were calculated as mean ± 1.96 SD of the differences.

Paired t-test and Wilcoxon Signed rank test were used to analyze differences between measurements obtained by the two keratometers and CHM. The correlation between results was probed using the Pearson coefficient. P-values less than 0.05 were considered as statistically significant. Bland-Altman plots were used to evaluate the agreement between measurements obtained by either instrument, and the CHM. In the Bland-Altman plots, differences between values obtained by the instruments and the CHM were plotted against mean values.

## RESULTS

Seventy eyes of 35 myopic patients including 10 male and 25 female subjects with mean age of 23.45 ± 1.55 years were included. None of the participants had previous history of keratorefractive surgery. Mean preoperative corneal thickness was 517.0 ± 11.2 (range, 496 to 548) microns and mean preoperative SE refractive error was −4.53 ± 1.30 D at 12 mm vertex distance and −4.28 ± 1.16 D at the corneal plane (Table 1). Mean preoperative keratometry was 45.95 ± 1.23 D with the IOLMaster and 46.32 ± 1.18 D with manual keratometry.

Postoperative Snellen uncorrected visual acuity was better than 9/10 in all eyes and 10/10 in 84.3% of eyes.

Table 2 details the correlation between keratometric measurements obtained by the two instruments and the CHM. The mean difference between CHM values and postoperative manual keratometry was smaller than the difference between CHM values and IOLMaster keratometry. The Pearson correlation coefficient was −0.042 for postoperative IOLMaster keratometry and CHM, and 0.484 for postoperative Javal keratometry and CHM; however, the lower and upper 0.95% LoA for manual keratometry and CHM was not narrower than the agreement between IOLMaster keratometry and CHM.

**Figure 1:**
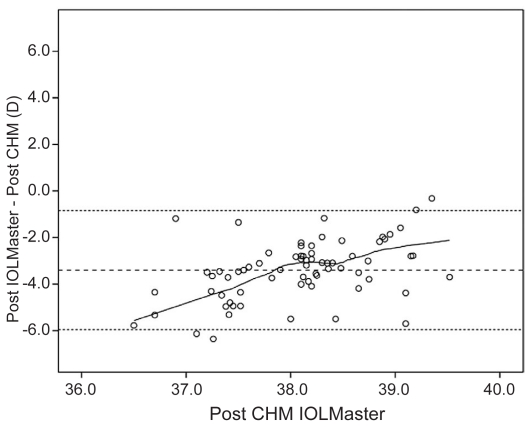
Bland-Altman plot of IOLMaster keratometric measurements versus CHM. The middle line represents the mean, and lines on either side represent the upper and lower 95% limits of agreement.

**Figure 2:**
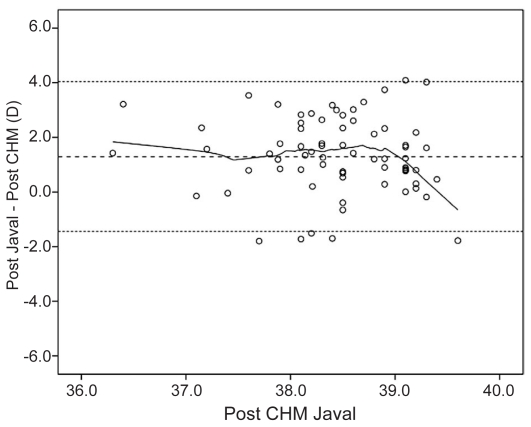
Bland-Altman plot of Javal manual keratometric measurements versus CHM. The middle line represents the mean, and lines on either side represent the upper and lower 95% limits of agreement.

Figures 1 and 2 show Bland-Altman plots of IOLMaster and manual keratometry versus CHM. These plots show no correlation between keratometry derived from these instruments and the CHM.

**Figure 3:**
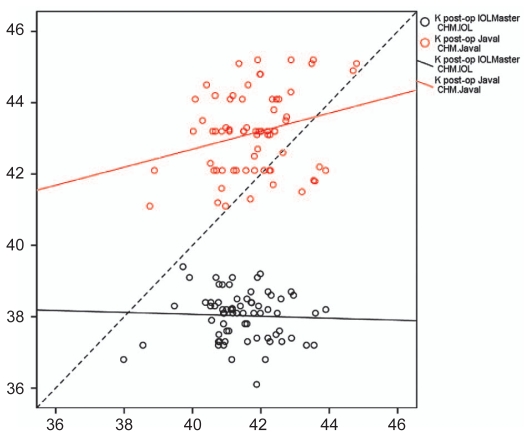
Scatter plot of IOLMaster and Javal manual keratometry against CHM measurements. The diagonal dotted line represents the line of equivalence.

Figure 3 shows scatter plots of IOLMaster and manual keratometric measurements against CHM. The lines for IOLMaster or manual keratometry values do not approach the 45° line, representing no correlation between the CHM and measured values by both instruments.

## DISCUSSION

An unfortunate consequence of keratorefractive surgery is inaccuracy in IOL power calculation.^8^,^9^ The present study compared keratometric measurements with two different instruments before and after PRK. Although the IOLMaster and a Javal type manual keratometry had high correlation preoperatively, the two devices demonstrated no correlation postoperatively and each of them yielded figures with large deviations from those obtained by the CHM.

In 2005, Schafer et al^5^ studied 58 eyes and compared keratometry after laser in situ keratomileusis (LASIK) with the IOLMaster to corneal topography and demonstrated a smaller mean deviation with the IOLMaster (38.94 ± 1.88 D vs. CHM: 38.35 ± 2.13 D) as compared to topography (39.84 ± 1.85 D vs. CHM: 38.86 ± 2.10 D).

Verhulst and Vrijghem^10^ in 2001 compared the Javal keratometer with the IOLMaster. The authors reported IOL power calculations using the IOLMaster to be easy to perform and result in excellent refractive outcomes. This is in contrast to our findings by which we observed no correlation between postoperative IOLMaster readings and the CHM, or between postoperative manual keratometry and the CHM. Apart from the two above-mentioned studies we could not find any other in the literature comparing the IOLMaster with Javal type manual keratometry.

Peter et al^11^, compared manual keratometry with videokeratography in 128 eyes and found that that neither manual keratometry using the Javal keratometer nor videokeratography was able to accurately reflect the changes in corneal power and refraction after PRK. They suggested that in reality keratometric values in the center of the cornea may be lower, since it is not taken into account by manual keratometry and videokeratography.

Savini et al^6^ reported that Pentacam measurements are not statistically different from corneal power values derived using the CHM in eyes that have previously undergone myopic excimer laser surgery and can be acceptably used, albeit with caution.

Several methods have been proposed to improve the accuracy of estimating corneal power in eyes that have undergone laser keratorefractive surgery. These approaches can be categorized according to whether or not they require knowledge of data acquired before the operation. Those that depend on preoperative data include the clinical history method^12^ (requires manifest refraction and corneal power values), the Feiz-Mannis method^12^ (requires manifest refraction and corneal power values), and a topographical method^13^ (requires manifest refraction). Methods that do not require preoperative data include contact lens over-refraction, adjusting corneal power using a correction factor, direct measurement using Orbscan topography, and a method proposed by Maloney.^14^,^15^

Although published studies suggest that the CHM is a helpful approach for calculating corneal power, the number of eyes studied is small and an unacceptably large number of refractive surprises has been reported.^16^–^18^ The CHM has been claimed to be the best method, but not infrequently it results in mediocre refractive outcomes. It has the additional disadvantage of requiring preoperative, operative, and stable postoperative data and can only be applied whenever refraction and keratometry before the procedure are available to the cataract surgeon.^19^–^21^

The Humphrey Zeiss IOLMaster may prove to be more accurate for determining functional axial length in extremely myopic eyes, because it measures the distance to the functional fovea. This, of course, requires fixation to achieve the best results, which may difficult in eyes with severe myopic degeneration.^14^ The IOLMaster measures 6 optical points within a hexagonal pattern in a 2.3 mm area at the air-tear film interface.^22^,^23^

The present study demonstrated that despite a high correlation between the IOLMaster and a Javal type manual keratometer preoperatively, these two devices were not comparable postoperatively. Both devices led to measurements significantly different from CHM-derived keratometry. The cause of this disagreement is probably multifactorial, and perhaps due to various corneal surface changes that occur after keratorefractive surgery. Available instruments may have different and unpredictable results from one person to another and results may differ from those obtained in previous studies. Significant refractive errors due to inaccurate IOL power may occur with each of these methods after keratorefractive surgery.

We believe that precise documentation of preoperative patient data and using the CHM remains the safest and most practical approach, until accuracy of IOL power calculation for eyes with previous keratorefractive surgery improves and hyperopic errors after cataract surgery in these eyes can be significantly reduced.

## Figures and Tables

**Table 1:** Pre- and postoperative keratometric measurements

	Preoperative	Postoperative	Difference		Percent of Change	P-value*

			Mean	95% CI		
IOL-Master	45.95 ± 1.23	38.03 ± 0.68	7.92	7.63 to 8.21	17.2	<0.001
Manual	46.32 ± 1.18	43.15 ± 1.13	3.16	2.81 to 3.51	6.8	<0.001

P-value*	<0.001	<0.001				

Sphere	−4.14 ± 1.30	0.08 ± 0.33	−4.22	−4.51 to −3.93	102.5	<0.001
Cylinder	−0.79 ± 0.38	0.30 ± 0.42	−1.09	−1.25 to −0.93	140.6	<0.001
SE	−4.53 ± 1.30	0.23 ± 0.44	−4.76	−5.07 to −4.45	105.6	<0.001
SE at corneal plane	−4.28 ± 1.16	0.23 ± 0.44	−4.51	−4.78 to −4.24	106	<0.001

CI, confidence interval; SE, spherical equivalent

* Wilcoxon Signed rank test.

**Table 2:** Correlation between keratometric measurements and the clinical history method

	Statistic	IOLMaster	Manual
K	mean ± SD	38.03 ± 0.68	43.15 ± 1.13
CHM	mean ± SD	41.43 ± 1.06	41.8 ± 1.16
K - CHM Difference	mean ± SD	−3.4 ± 1.3	1.3 ± 1.4
	95% CI	−3.71 to −3.1	1.02 to 1.68
	P-value	0.000	0.000
Correlation	R	−0.042	0.484
	P-value	0.728	<0.001
LoA		−5.95 to −0.85	−1.44 to 4.04

K, keratometry measurement; CHM, clinical history method; SD, standard deviation; CI, confidence interval; R, Pearson correlation; LoA, limit of agreement

* Paired t-test
